# Bevacizumab for Patients with Recurrent Gliomas Presenting with a Gliomatosis Cerebri Growth Pattern

**DOI:** 10.3390/ijms18040726

**Published:** 2017-03-29

**Authors:** Michael C. Burger, Iris C. Mildenberger, Marlies Wagner, Michel Mittelbronn, Joachim P. Steinbach, Oliver Bähr

**Affiliations:** 1 Dr. Senckenberg Institute of Neurooncology, Goethe University, 60528 Frankfurt, Germany; iris.mildenberger@umm.de (I.C.M.); joachim.steinbach@med.uni-frankfurt.de (J.P.S.); oliver.baehr@med.uni-frankfurt.de (O.B.); 2 Institute of Neuroradiology, Goethe University, 60528 Frankfurt, Germany; Marlies.Wagner@kgu.de; 3 Luxembourg Centre of Neuropathology (LCNP), 3555 Dudelange, Luxembourg; michel.mittelbronn@lns.etat.lu; 4 Laboratoire National de Santé, 3555 Dudelange, Luxembourg; 5 Luxembourg Centre for Systems Biomedicine (LCSB), University of Luxembourg, 4362 Esch-sur-Alzette, Luxembourg; 6 NORLUX Neuro-Oncology Laboratory, Department of Oncology, Luxembourg Institute of Health (L.I.H.), 1526 Luxembourg, Luxembourg; 7 Institute of Neurology (Edinger Institut), Goethe University, 60528 Frankfurt, Germany

**Keywords:** primary brain tumors, glioma, glioblastoma, gliomatosis cerebri growth pattern, anti-angiogenic therapy, bevacizumab

## Abstract

Bevacizumab has been shown to improve progression-free survival and neurologic function, but failed to improve overall survival in newly diagnosed glioblastoma and at first recurrence. Nonetheless, bevacizumab is widely used in patients with recurrent glioma. However, its use in patients with gliomas showing a gliomatosis cerebri growth pattern is contentious. Due to the marked diffuse and infiltrative growth with less angiogenic tumor growth, it may appear questionable whether bevacizumab can have a therapeutic effect in those patients. However, the development of nodular, necrotic, and/or contrast-enhancing lesions in patients with a gliomatosis cerebri growth pattern is not uncommon and may indicate focal neo-angiogenesis. Therefore, control of growth of these lesions as well as control of edema and reduction of steroid use may be regarded as rationales for the use of bevacizumab in these patients. In this retrospective patient series, we report on 17 patients with primary brain tumors displaying a gliomatosis cerebri growth pattern (including seven glioblastomas, two anaplastic astrocytomas, one anaplastic oligodendroglioma, and seven diffuse astrocytomas). Patients have been treated with bevacizumab alone or in combination with lomustine or irinotecan. Seventeen matched patients treated with bevacizumab for gliomas with a classical growth pattern served as a control cohort. Response rate, progression-free survival, and overall survival were similar in both groups. Based on these results, anti-angiogenic therapy with bevacizumab should also be considered in patients suffering from gliomas with a mainly infiltrative phenotype.

## 1. Introduction

Bevacizumab (BEV) is a humanized monoclonal antibody targeting VEGF-A, which is being secreted by malignant gliomas [1]. VEGF-A induces neovascularization by the formation of a dysfunctional vascular system, and an increase in permeability of the physiologically tight blood–brain barrier [2]. Increased vascular permeability results in pronounced extracellular edema, which is frequently seen in malignant gliomas [3]. By blocking VEGF, neo-angiogenesis is reduced, and the highly abnormal tumor vasculature partially normalizes to a more physiologically condition [4,5]. The peritumoral edema is decreased through the reduction of vascular permeability [6]. In this initial phase, normalization of the highly dysfunctional neovasculature leads to an improvement of tumor perfusion and oxygenation as well [7]. However, with proceeding regression of tumor vasculature, the supply with oxygen and glucose gets increasingly insufficient [8,9].

In mouse xenograft models BEV treatment led to an accumulation of the hypoxia markers HIF-1α and carboanhydrase IX (CAIX) [10,11,12]. The glioma xenografts evolved to a more invasive phenotype and increasingly invaded adjacent brain tissue not yet affected by the hypoxic pressure [13,14]. However, these preclinical results were not confirmed in patients with malignant brain tumors. It has been shown that, in glioblastoma (GBM), anti-angiogenic therapy does not lead to a more invasive phenotype to evade the strong selection pressure with hypoxia and low glucose concentration caused by BEV treatment [15].

Invading glioma cells at least initially do not depend on tumor neovasculature. Therefore, it seems questionable if anti-angiogenic therapy with BEV may have an effect in patients suffering from gliomas with a gliomatosis cerebri growth pattern. In these patients, tumor cells are characterized by a very invasive phenotype and poor or no neovascularization. These characteristics may render the tumor cells more capable of evading the therapeutic effect of BEV therapy.

Due to these uncertainties, BEV is often withheld from glioma patients with a mainly diffusely infiltrating tumor phenotype in clinical practice, even if there are no further therapeutic options available. In contrast, in glioma patients with a mainly solid phenotype, BEV is usually applied when all other established treatment options have failed. In this situation, physicians and their patients commonly face the decision between BEV treatment and cessation of tumor-specific therapy (“best supportive care”). Based on the considerations that tumors with a gliomatosis-like growth pattern show more non-angiogenic growth and thereby might be less accessible with a VEGF inhibiting strategy, response rate, progression-free survival (PFS), and overall survival (OS) should be lower in these patients. Therefore, we performed a retrospective data analysis of all patients with a gliomatosis cerebri growth pattern treated with BEV alone or in combination at our institution compared to a matched control cohort with a classical growth pattern.

In the updated “WHO Classification of Tumours of the Central Nervous System” from May 2016, gliomas with a mainly diffuse infiltrative phenotype involving at least three lobes were classified as having a “gliomatosis cerebri growth pattern”. In the previous WHO classification, gliomatosis cerebri was thought to be a distinct nosological glioma entity, but it is now considered only a pattern of exceptionally widespread involvement affecting three or more cerebral lobes. DNA-based molecular profiling did not find any typical gliomatosis cerebri pattern. Instead, these analyses showed that gliomatosis cerebri comprises a heterogeneous group of diffuse gliomas with DNA methylation and copy number profiles characteristic for the conventional molecularly defined glioma entities [16].

## 2. Results

**Figure 1:**
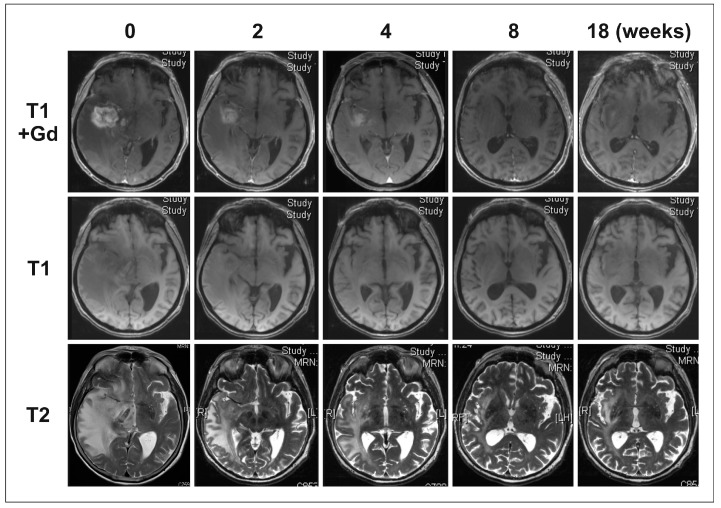
Exemplary magnetic resonance imaging (MRI) of Patient 7: contrast enhancing lesion. T1 sequences with and without Gadolinium contrast enhancer and T2 sequences were obtained at Weeks 0, 2, 4, 8 and 18 after BEV therapy initiation. After BEV therapy initiation contrast enhancing and T2 hyperintensive alterations decreased. At Week 18, a small contrast-enhancing alteration formed and the T2 hyperintensive alterations were increasing.

**Figure 2:**
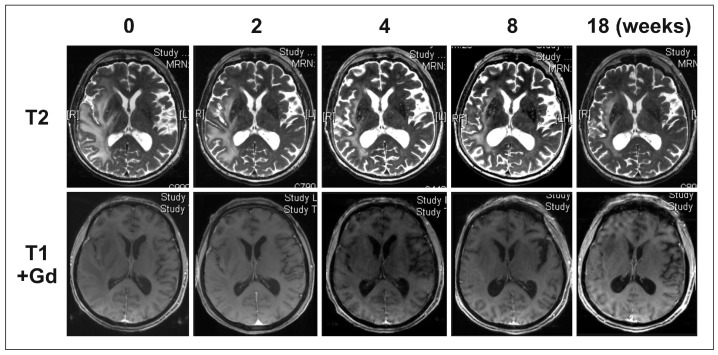
Exemplary magnetic resonance imaging (MRI) of Patient 7: non-contrast enhancing lesion. T2 sequences and T1 sequences after intravenous gadolinium administration were obtained at Weeks 0, 2, 4, 8 and 18 after BEV therapy initiation. While throughout the observation period no contrast enhancement was observed on this level, the T2 sequences from Week 18 show non-enhancing progressive disease.

**Figure 3:**
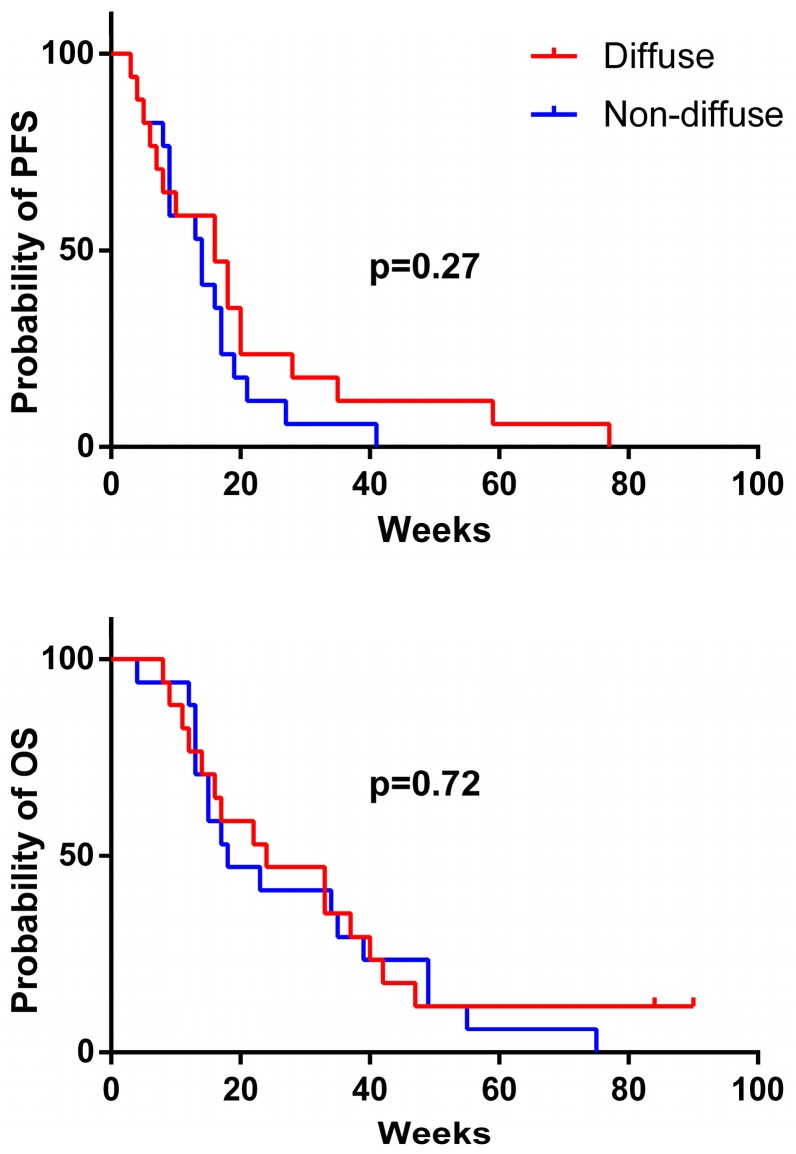
Progression-free (PFS) and overall survival (OS). No significant difference in PFS and OS from patients with a gliomatosis cerebri growth pattern (diffuse) compared to patients with a predominantly solid tumor phenotype (non-diffuse) was observed.

In the patient group with gliomatosis-like disease, the best response was a partial response (PR) in eight patients, a stable disease (SD) in three patients, and a progressive disease (PD) in six patients (exemplary for Patient 7, see Figure 1 and Figure 2). In the non-gliomatosis-like control group, the best response was PR in seven patients, SD in two patients, and PD in eight patients. Progression-free survival (PFS) and overall survival (OS) did not differ significantly between patients with gliomatosis-like and non-gliomatosis-like disease (Figure 3). For patients with gliomatosis-like disease, median PFS was 16 weeks, and 14 weeks for patients with non-gliomatosis-like disease (see Table 1 and Table 2). Median OS was 24 weeks for patients with gliomatosis-like disease, and 18 weeks for patients with non-gliomatosis-like disease. In the patients with gliomatosis-like disease, Karnofsky performance score (KPS) improved under therapy in six patients, stabilized in six patients, and deteriorated in five patients. In the control group with non-gliomatosis-like disease, KPS improved in four patients, stabilized in eight patients, and deteriorated in five patients. Of the patients with gliomatosis-like disease, steroid intake could be reduced after BEV therapy initiation in six patients, was stable in eight patients, and had to be escalated in three patients. In the control group with non-gliomatosis-like disease, steroid intake was reduced in ten patients, was stable in five patients, and had to be increased in two patients (see Table 1 and Table 2). Interestingly, the effect on contrast-enhancing and non-contrast-enhancing tumor areas was similar in most patients (see Table 1 and Table 2).

## 3. Discussion

This patient series shows that a diffuse infiltrating tumor phenotype does not impair response to BEV treatment. We were able to confirm the finding of the exploratory post-hoc evaluation of the AVAglio trial that the effect of BEV on PFS is similar in patients with gliomatosis-like and non-gliomatosis-like disease in patients with glioblastoma (GBM) [17]. While all patients examined in the AVAglio data were selected patients with GBM qualifying for participation in a therapy study, our data are derived from a much more diverse and heavily pretreated patient group with gliomas WHO°II-IV. In particular, a focal GBM histology is rather uncommon in patients suffering from gliomas with a gliomatosis-like disease pattern. In the NOA-05 trial, which defines the treatment of these patients, only 11% were characterized by a focal GBM histology. In 63% of the patients the diagnosis was based on a focal glioma WHO°II histology (astrocytoma 57%, oligoastrocytoma 6%) and in 26% of the patients on a glioma WHO°III histology (anaplastic astrocytoma 20%, anaplastic oligoastrocytoma 6%) [18]. In our patient series, the proportion of patients was still skewed towards a focal GBM histology due to the established treatment decision for BEV, which was based on the presence of contrast-enhancing tumor areas. In our series, the diagnosis was based in 41% on a focal glioma WHO°II histology, and in 18% on a focal glioma WHO°III histology. Furthermore, in the AVAglio study, tumors were classified as “diffuse (infiltrative)” if there was “extensive hypersignal/hyperintensity on T2-w or FLAIR images” [17], regardless of how many cerebral lobes were affected. Therefore, some patients may have been included into this analysis, where a “gliomatosis cerebri growth pattern” as defined by the previous WHO classification was not present. Summarizing, the results of our patient series were similar to those of the AVAglio trial, although our patient series might better represent the “real-life situation”.

The proportion of patients with an IDH-mutation in this series was rather low. Two out of 12 patients tested (1 of 4 low-grade gliomas tested) with gliomatosis-like disease were positive for an IDH1R132H mutation (see Table 3). In patients with gliomas and a gliomatosis cerebri growth pattern, IDH mutations are less frequent than in gliomas with a more solid phenotype, even in low-grade gliomas [19]. However, IDH-mutations were rare in the control cohort as well (1 out of 12 patients tested, 0 out of 3 low-grade gliomas tested; see Table 4). This discrepancy to published studies (i.e., 48% mIDH1R132H mutations in the NOA-05 trial [18]) may have several reasons. One reason may be the high proportion of patients with GBM and anaplastic astrocytomas in our series. Furthermore, the patients in our series were preselected by the established decision for BEV therapy, which was influenced by the presence of contrast enhancing lesions. Possibly the more aggressive growth of IDH wildtype tumors and the tendency to earlier relapses in these patients also led to a more aggressive therapeutic approach, which favored the selection of a controversial therapy like BEV. However, given the low number of patients tested for mIDH1R132H, the results should be interpreted cautiously.

Interestingly, the effects on contrast-enhancing and non-contrast-enhancing tumor areas were similar. The reasons for this finding are not yet clear. The BEV effect on T2 hyperintensive but non-contrast-enhancing tumor areas was beyond what could be expected by sole edema reduction. While direct effects of VEGF inhibition on glioma cells are well-known [20,21,22], the clinical relevance of these observations is still unclear.

Delaying the development of new contrast-enhancing tumor nodes in T2 hyperintensive areas by BEV therapy may also prolong PFS. However, there are still no data on the significance of this rather hypothetical mechanism.

However, the reservation must be made that a comparison of patients with gliomatosis-like disease treated with BEV to those who did not receive BEV was not feasible. The patients of our series were preselected, as the decision for BEV therapy was made for all patients. BEV therapy was initiated when no further established therapy regime seemed promising. Further arguments for BEV therapy initiation were extensive tumor masses, imaging indicative of focal malignization and resistance to steroid therapy in patients with a relatively good clinical condition (KPS ≥ 60%). In those patients where BEV therapy was omitted, i.e., due to a poor clinical condition (KPS < 60%), tumor-specific therapy was terminated. Therefore, we could not establish a suitable matched control cohort of patients without BEV therapy. A direct comparison of patients with gliomatosis-like disease treated with BEV to those who received “best supportive care” only would be desirable. Such a trial should be designed as a prospective randomized trial.

Several studies have shown that BEV treatment in patients with glioblastoma (GBM) prolongs PFS and delays neurological deterioration [17,23,24,25]. Delaying clinical progression, cognitive and functional decline and deterioration of the quality of life is of high importance to patients and their relatives [26]. Therefore, although several studies have shown no effect on OS in GBM [17,23,24,25], BEV is still widely applied in the relapse situation, especially in patients where no further therapeutic alternatives are available. In our series, a significant proportion of patients both with gliomatosis-like and non-gliomatosis-like diseases even improved clinically after initiation of BEV treatment. Our patient series as well as the AVAglio study has shown that the response rates, PFS, and OS are similar in patients suffering from gliomas with gliomatosis-like and non-gliomatosis-like disease patterns. Therefore, BEV should not be withheld from patients only because of a mainly diffuse infiltrative growth pattern.

## 4. Patients and Methods

Between April 2008 and March 2016, a total of 164 patients with glioblastoma (GBM), 27 patients with anaplastic astrocytoma (AA), 9 patients with anaplastic oligodendroglioma (AO), and 23 patients with diffuse astrocytoma (A) were treated with BEV at our center. The main inclusion criterion was a mainly diffuse infiltrating and mostly non-enhancing tumor involving at least three lobes. We identified 17 consecutive patients with gliomas showing a diffuse infiltrative tumor growth pattern. All patients were treated with BEV (10 mg/kg IV every other week) between April 2008 and March 2016 in combination with lomustine (CCNU) or irinotecan, or as a single agent on an individual basis (see Table 3).

In 7 patients, the histology was GBM, in 2 patients AA, in 1 patient AO, and in 7 patients A. A matched the control cohort of patients with a non-gliomatosis cerebri tumor phenotype treated with BEV during the same time interval at our institution was selected based on histology, pretreatment, and Karnofsky performance score (KPS) at the time of BEV therapy initiation (see Table 4). As none of the patients with gliomatosis-like growth pattern had received total resection at initial diagnosis, gross total resections were excluded from the control group as well. Progression-free survival and overall survival were estimated using the Kaplan–Meier method. Significance of the survival analyses was calculated using the Log-rank (Mantel–Cox) test.

Patient characteristics in the gliomatosis-like and the non-gliomatosis-like groups were similar (see Table 5). However, due to the inherent tumor distribution, biopsy only as compared to partial resection at initial diagnosis was more common in the gliomatosis-like group than in the non-gliomatosis-like group (59% vs. 35%). The molecular testing results (i.e., MGMT methylation and IDH1 mutation status) were similar in both groups.

In all patients of this series, the rationale for BEV therapy initiation was progression of the contrast-enhancing tumor areas.

### 4.1. MGMT Promoter Methylation Status Assessment

Two to six tumor specimens showing the largest amount of vital tumor tissue were selected for methylation-specific polymerase chain reaction (MSP). Four slides with a 10 µm thickness were cut from each paraffin block. Slides were deparaffinized using xylene and 2 × 96% alcohol. DNA-Isolation was performed using DNeasy Blood & Tissue Kit (Quiagen, Hilden, Germany). Nucleic acid concentration was determined by a UV spectrophotometric analysis using Nanodrop 1000 Spectrophotometer. DNA was treated with sodium bisulfite using the EZ DNA Methylation-Gold Kit (Zymo Research, Irvine, CA, USA). PCR run was performed on the Thermocycler T3000 (Biometra, Göttingen, Germany). For PCR, 2 µL of sodium bisulfite-pretreated DNA was amplified by the following primer sets: (I) *MGMT*-methylated forward primer: 5′-GTTTTTAGAACGTTTTGCGTTTCGAC-3′; (II) *MGMT*-methylated reverse primer: 5′-CACCGTCCCGAAAAAAAACTCCG-3′; (III) *MGMT*-unmethylated forward primer: 5′-TGTGTTTTTAGAATGTTTTGTGTTTTGAT-3′; (IV) *MGMT*-unmethylated reverse primer: 5′-CTACCACCATCCCAAAAAAAAACTCCA-3′. For the methylated *MGMT* sequence, a 122 bp fragment is expected while the amplification of the unmethylated *MGMT* sequence results in a fragment of 129 bp. In each MSP run, DNA from the glioma cell line, LNT-229 was used as a positive control for a methylated *MGMT* promoter, DNA from healthy volunteer donors were used as a positive control for an unmethylated *MGMT* promoter status, and H_2_O was used as a negative control. After MSP, 20 µL of each sample was loaded on a 2% agarose-gel at 120 V for 35 min.

### 4.2. Analysis of 1p and 19q Loss by Fluorescence In Situ Hybridization (FISH)

Samples were analyzed by fluorescence in situ hybridization (FISH) to assess for 1p and 19q deletions. The two-color FISH assay was performed on sections 3 µm thick using a mixed 1p36/1q25 dual color probe and 19p13/19q13 dual color probe set (ZytoLight SPEC, Cat. No. Z-2075 and Z-2076, Zyto-Vision, Bremerhaven, Germany). The Histology Accessory FISH Kit (Dako, Glostrup, Denmark) was used for slide pre-treatment, probe hybridization, and post-hybridization processing. Nuclei were counterstained with DAPI/Antifade-Solution (ZytoVision). Fluorescent signals were analyzed using an Olympus BX50 fluorescent microscope with the appropriate filters (Olympus, Hamburg, Germany). Samples displaying sufficient FISH efficiency (≥80% fluorescent nuclei) were evaluated. Signals were scored in at least 100 non-overlapping, intact nuclei. Deletions of 1p or 19q were defined by samples with over 50% of the tumor nuclei containing only one signal.

### 4.3. mIDH1R132H Immunohistochemistry

The immunostainings for mutated IDH1R132H were performed using standard diagnostic protocols and the DiscoveryXT immunohistochemistry system (Ventana, Strasbourg, France). The following antibody was used: monoclonal mouse anti-human mIDH1R132H (dilution 1:50; clone H09, DIANOVA GmbH, Hamburg, Germany).

Our institutional review board approved this retrospective study, and patients gave their written consent for scientific work with clinical data including MRI scans (ethics committee at the University Hospital Frankfurt; reference number 04/09-SNO 22 January 2009).

## Figures and Tables

**Table 1:** Outcome of the patients with a diffuse growth pattern.

Pat. No.	Combination Therapy	Karnofsky Performance Score (KPS)		Steroid Intake (mg of Dexamethasone per Day)		Best Response (RANO Criteria)	CE	NCE	PFS (Weeks)	OS (Weeks)
		at Start of Therapy	Development under Therapy	at Start of Therapy	under Therapy					
1	none	80	+10	0	0	PR	+	+	28	37
2	none	70	0	0	0	SD	0	0	20	40
3	none	60	+20	16	0	PR	+	+	16	33
4	Iri	90	−10	8	12	PD	−	−	4	11
5	none	70	−10	2	4	PD	0	−	5	9
6	none	70	0	4	4	PD	−	−	6	17
7	CCNU	60	+10	16	1	PR	+	+	18	22
8	CCNU	80	−10	4	2	PD	−	0	7	16
9	Iri	80	0	0	0	SD	0	0	18	24
10	none	80	+10	0	0	SD	0	0	16	33
11	none	60	−10	3	12	PD	0	0	8	12
12	CCNU	70	+10	2	0	PR	+	+	59	n.r.
13	CCNU	60	−10	8	8	PD	0	−	3	8
14	Iri	80	+10	0	0	PR	+	+	10	14
15	none	90	0	0	0	PR	+	+	20	42
16	none	60	0	6	0	PR	+	0	35	47
17	none	80	0	1	0	PR	+	0	77	n.r.

+ = sum of product of diameters (SPD) decreased by ≥50%; − = SPD increased by ≥25%; 0 = SPD <50% decrease to <25% increase; CCNU = lomustine; CE = contrast-enhancing tumor areas as measured from the MRI with best response; Iri = irinotecan; NCE = non-contrast-enhancing tumor areas as measured from the MRI with best response; n.r. = not reached; OS = overall survival; PD = progressive disease; PFS = progression free survival; PR = partial response; SD = stable disease.

**Table 2:** Outcome of the patients with a non-diffuse growth pattern.

Pat. No.	Combination Therapy	Karnofsky Performance Score (KPS)		Steroid Intake (mg of Dexamethasone per Day)		Best Response (RANO Criteria)	CE	NCE	PFS (Weeks)	OS (Weeks)
		at Start of Therapy	Development under Therapy	at Start of Therapy	under Therapy					
C1	none	80	0	4	0.5	SD	0	+	19	35
C2	none	70	0	4	0	SD	0	+	41	55
C3	Iri	60	+20	4	0	PR	+	+	16	39
C4	none	90	−20	8	12	PD	−	−	9	15
C5	CCNU	70	+10	6	2	PR	+	+	21	49
C6	none	70	0	2	2	PR	+	+	17	34
C7	none	60	+10	4	4	PR	+	+	14	18
C8	CCNU	70	0	4	2	PR	+	0	17	23
C9	Iri	80	−10	4	4	PD	−	−	9	12
C10	none	90	−10	8	12	PD	0	−	5	13
C11	none	60	0	16	4	PD	−	−	4	13
C12	CCNU	70	+10	2	0	PR	+	0	14	75
C13	none	60	0	4	0	PR	+	0	27	49
C14	Iri	80	−10	0	0	PD	0	+	8	13
C15	none	90	0	0	0	PD	−	−	13	15
C16	none	60	−10	4	0	PD	−	−	3	4
C17	CCNU	70	0	4	0	PD	0	−	9	17

+ = sum of product of diameters (SPD) decreased by ≥50%; − = SPD increased by ≥25%; 0 = SPD <50% decrease to <25% increase; CCNU = lomustine; CE = contrast-enhancing tumor areas as measured from the MRI with best response; Iri = irinotecan; NCE = non-contrast-enhancing tumor areas as measured from the MRI with best response; OS = overall survival; PFS = progression free survival.

**Table 3:** Characteristics of patients with a diffuse growth pattern.

Pat. No.	Age	Gender	Histology	Genetic Testing	Pretreatment
1	34	F	GB	MGMT meth, mIDH1R132H+	TMZ 7/14, WBXRT, reS, CCNU/VM26
2	43	F	GB	MGMT unmeth, mIDH1R132H−	pS, IFXRT, Enza, TMZ 7/14, CCNU/VM26
3	43	M	GB	n.d.	pS, IFXRT, TMZ, reS, TMZ 7/14
4	46	M	GB	MGMT meth, mIDH1R132H−	PC, CCNU, TMZ-IFXRT
5	58	M	GB	MGMT unmeth, mIDH1R132H−	WBXRT, CCNU/TMZ
6	63	F	GB	MGMT unmeth, mIDH1R132H−	pS, TMZ-IFXRT, TMZ, reS, TMZ 7/14, CCNU/VM26
7	65	F	GB	MGMT unmeth, mIDH1R132H−	TMZ-IFXRT, TMZ
8	54	M	AA	MGMT ic, mIDH1R132H−	TMZ, TMZ-IFXRT, CCNU/VM26
9	70	M	AA	mIDH1R132H−	TMZ 7/14, PC, IFXRT
10	51	M	AO	n.d.	TMZ, TMZ 7/14, TMZ-IFXRT, CCNU
11	27	F	A	mIDH1R132H+	IFXRT, TMZ, CCNU
12	37	F	A	mIDH1R132H−	IFXRT, TMZ 7/14, TMZ-IFXRT
13	42	M	A	mIDH1R132H−, no 1p/19q codeletion	pS, TMZ, TMZ-IFXRT, CCNU/VP16
14	42	M	A	n.d.	TMZ, PC, WBXRT
15	53	F	A	n.d.	pS, TMZ-IFXRT, TMZ 7/14
16	60	F	A	n.d.	pS, TMZ, TMZ 7/14, WBXRT
17	67	M	A	MGMT ic, mIDH1R132H−, no 1p/19q codeletion	IFXRT, PCV

A = Diffuse Astrocytoma WHO°II; AA = Anaplastic Astrocytoma WHO°III; AO = Anaplastic Oligodendroglioma WHO°III; CCNU = lomustine; CCNU/TMZ = lomustine/temozolomide; CCNU/VM26 = lomustine/teniposide; CCNU/VP16 = lomustine/etoposide; Enza = Enzastaurin; GB = Glioblastoma WHO°IV; IFXRT = involved field radiation therapy; MGMT ic = result for MGMT promotor methylation testing inconsistent; MGMT meth = MGMT promoter hypermethylation; MGMT unmeth = no MGMT promotor hypermethylation; mIDH1R132H+ = immunoreactivity with a mIDH1R132H specific antibody; n mIDH1R132H− = no immunoreactivity with a mIDH1R132H specific antibody; n.d. = not determined; PC = procarbacine/lomustine; PCV = procarbacine/lomustine/vincristine; pS = surgery with partial resection; reS = relapse surgery; TMZ = temozolomide 5/28; TMZ 7/14 = dose dense temozolomide (one week on/one week off); TMZ-IFXRT = involved field radiation therapy with concomitant temozolomide WBXRT = whole brain radiation therapy.

**Table 4:** Characteristics of the patients of the control group with a non-diffuse growth pattern.

Pat. No.	Age	Gender	Histology	Genetic Testing	Pretreatment
C1	31	M	GBM	mIDH1R132H−	pS, TMZ-IFXRT, TMZ 7/14, TMZ, reS, CCNU
C2	44	F	GBM	MGMT unmeth, mIDH1R132H+	pS, TMZ-IFXRT, TMZ, TMZ 7/14
C3	43	M	GBM	MGMT meth, mIDH1R132H−	pS, TMZ-IFXRT, TMZ, reS, TMZ 7/14
C4	47	M	GBM	MGMT unmeth, mIDH1R132H−	TMZ-IFXRT, TMZ, TMZ 7/14, CCNU
C5	58	F	GBM	MGMT meth, mIDH1R132H−	TMZ-IFXRT, TMZ
C6	62	M	GBM	MGMT unmeth, mIDH1R132H−	pS, TMZ-IFXRT, TMZ, TMZ 7/14, reS, CCNU
C7	72	M	GBM	MGMT unmeth, mIDH1R132H−	TMZ-IFXRT; TMZ
C8	59	F	AA	mIDH1R132H−	TMZ-IFXRT, TMZ, CCNU
C9	59	F	AA	mIDH1R132H−	IFXRT, TMZ, CCNU
C10	52	F	AO	n.d.	pS, TMZ-IFXRT, TMZ, TMZ 7/14, CCNU
C11	43	M	A	n.d.	pS, TMZ, IFXRT, CCNU
C12	34	M	A	mIDH1R132H−	pS, IFXRT, TMZ
C13	44	M	A	MGMT unmeth, mIDH1R132H−, no 1p/19q codeletion	pS, TMZ-IFXRT, TMZ, CCNU/VM26
C14	46	M	A	n.d.	pS, IFXRT, TMZ, CCNU
C15	46	F	A	n.d.	pS, IFXRT, TMZ 7/14
C16	42	M	A	n.d.	pS, IFXRT, TMZ, TMZ 7/14
C17	55	F	A	mIDH1R132H−, no 1p/19q codeletion	IFXRT, CCNU/VM26

A = Diffuse Astrocytoma WHO°II; AA = Anaplastic Astrocytoma WHO°III; AO = Anaplastic Oligodendroglioma WHO°III; CCNU = lomustine; CCNU/VM26 = lomustine/teniposide; GB = Glioblastoma WHO°IV; IFXRT = involved field radiation therapy; MGMT meth = MGMT promoter hypermethylation; MGMT unmeth = no MGMT promotor hypermethylation; mIDH1R132H+ = immunoreactivity with a mIDH1R132H specific antibody; mIDH1R132H− = no immunoreactivity with mIDH1R132H specific antibodies; n.d. = not determined; pS = surgery with partial resection; reS = relapse surgery; TMZ = temozolomide 5/28; TMZ 7/14 = dose dense temozolomide (one week on/one week off); TMZ-IFXRT = involved field radiation therapy with concomitant temozolomide WBXRT = whole brain radiation therapy.

**Table 5:** Comparison of patient characteristics.

Patient Characteristics	Gliomatosis-Like	Non-Gliomatosis-Like
Female/male patients	8/9	7/10
Median patients’ age at initiation of BEV therapy (years)	51	46
Partial surgery/biopsy at diagnosis	6/9	11/6
Median previous chemotherapy lines	2	2
Median KPS score at initiation of BEV therapy	70	70
Median time between diagnosis and initiation of BEV therapy (months)	36	33
Median steroid intake at initiation of BEV therapy (mg of dexamethasone per day)	2	4

BEV = bevacizumab; KPS = Karnofsky performance score.
